# Functional analysis of CqPORB in the regulation of chlorophyll biosynthesis in *Chenopodium quinoa*

**DOI:** 10.3389/fpls.2022.1083438

**Published:** 2022-12-12

**Authors:** Chao Li, Minyuan Ran, Jianwei Liu, Xiaoxiao Wang, Qingbing Wu, Qiang Zhang, Jing Yang, Feng Yi, Heng Zhang, Jian-Kang Zhu, Chunzhao Zhao

**Affiliations:** ^1^ Shanghai Center for Plant Stress Biology, CAS Center for Excellence in Molecular Plant Sciences, Chinese Academy of Sciences, Shanghai, China; ^2^ University of the Chinese Academy of Sciences, Beijing, China; ^3^ Bright Agricultural Development (Group) Co., Ltd., Shanghai, China; ^4^ National Key Laboratory of Plant Molecular Genetics, Shanghai Center for Plant Stress Biology, CAS Center for Excellence in Molecular Plant Sciences, Chinese Academy of Sciences, Shanghai, China; ^5^ Institute of Advanced Biotechnology and School of Life Sciences, Southern University of Science and Technology, Shenzhen, China

**Keywords:** protochlorophyllide oxidoreductase (POR), quinoa (*Chenopodium quinoa* Willd), protochlorophyllide, chlorophyll, grana stacks

## Abstract

Protochlorophyllide oxidoreductase (POR) plays a key role in catalyzing the light-dependent reduction of protochlorophyllide (Pchlide) to chlorophyllide (Chlide), and thus promotes the transit from etiolated seedlings to green plants. In this study, by exploring ethyl methanesulfonate (EMS)-mediated mutagenesis in *Chenopodium quinoa* NL-6 variety, we identified a mutant *nl6-35* that displays faded green leaf and reduced chlorophyll (Chl) and carotenoid contents. Bulk segregant analysis (BSA) revealed that a mutation in *CqPORB* gene is genetically associated with the faded green leaf of the *nl6-35* mutant. Further study indicates that the *nl6-35* mutant exhibits abnormal grana stacks and compromised conversion of Pchlide to Chlide upon illumination, suggesting the important role of *CqPORB* in producing photoactive Pchlide. Totally three *CqPOR* isoforms, including *CqPORA*, *CqPORA-like*, and *CqPORB* are identified in NL-6 variety. Transcriptional analysis shows that the expression of all these three *CqPOR* isoforms is regulated in light- and development-dependent manners, and in mature quinoa plants only *CqPORB* isoform is predominantly expressed. Subcellular localization analysis indicates that CqPORB is exclusively localized in chloroplast. Together, our study elucidates the important role of CqPORB in the regulation of Chl biosynthesis and chloroplast development in quinoa.

## Introduction

*Chenopodium quinoa* Willd. (quinoa) is an annual pseudocereal crop that belongs to Amaranthaceae family, and this family also encompasses other economically important species such as spinach (*Spinacia olereaceae* L.) and sugar beet (*Beta vulgaris* L.) (Mizuno et al., 2020). Quinoa originated from the Andean region of South America and is now cultivated worldwide, especially since the declaration of the year 2013 as the International Year of Quinoa by the United Nations Food and Agricultural Organization (FAO) (Mizuno et al., 2020). Compared with the major staple cereal crops, such as rice, wheat, oat, and maize, that lack enough lysine for the daily requirement of human being, quinoa seeds contain a more balanced amino acid profile, particularly for the rich of lysine, histidine, and methionine (Dakhili et al., 2019). In addition, quinoa is rich in protein, starch, lipid, a variety of vitamins, minerals, and some health-beneficial phytochemicals (Graf et al., 2016; Cai and Gao, 2020). Due to its exceptional nutritional value, quinoa is considered as a “perfect and strategic food” by FAO (Dakhili et al., 2019), and it was selected as desirable food for astronauts in space by the National Aeronautics and Space Administration (NASA) of the United States (Yasui et al., 2016). In addition to its high nutritional value, quinoa is regarded as a halophyte that tolerates multiple abiotic stresses, including high salinity, drought, and low temperature, which makes it a promising crop to grow in marginal areas (Hariadi et al., 2011; Böhm et al., 2018). In recent years, three quinoa reference genomes from the accessions of Kd, QQ74, and Real have been independently sequenced and assembled (Yasui et al., 2016; Jarvis et al., 2017; Zou et al., 2017), which opens a door to study the agronomic traits of quinoa in a genome-wide and molecular levels.

Although quinoa was domesticated over 7,000 years ago in the Andean region of South America (Dillehay et al., 2007), the average yield of quinoa is still relatively low and highly dependent on climate and growth conditions. Therefore, yield improvement is currently an urgent issue for quinoa breeding to meet the increasing of global demand for quinoa. Photosynthesis is an important biological process that enables plants to convert sunlight energy to carbohydrates, and thus promotes plant growth and yield (Eberhard et al., 2008). Engineering of photosynthesis machinery is a feasible and useful strategy to improve crop yield, which has been reported in several studies (Long et al., 2015; Chen et al., 2020). Chloroplast is the cellular organelle in plants that serves as the site for photosynthesis, and chlorophyll (Chl) biosynthesis occurred in chloroplast is essential for plants to execute photosynthesis. Chl is the most abundant pigment in land plants and algae that is indispensable for light absorption and energy transfer during photosynthesis (Allen et al., 2011; Wang and Grimm, 2021). Chl biosynthesis has been studied for decades, and it has been known that enzymes, including magnesium chelatase (MgCh), Mg-protoporphyrin IX methyltransferase (MgMT), Mg-protoporphyrin IX monomethyl ester cyclase (MgCy), protochlorophyllide oxidoreductase (POR), divinylchlorophyllide reductase (DVR), and Chl synthase, are involved in this process (Tanaka and Tanaka, 2007). Chl biosynthesis is tightly controlled in response to environmental light signal. In darkness, Chl biosynthesis is halted at the step of protochlorophyllide (Pchlide), which is the precursor of chlorophyllide (Chlide), while upon illumination, Pchlide is converted to Chlide, and subsequently Chl is synthesized (Reinbothe et al., 2010). The conversion of Pchlide to Chlide is considered as a key step for de-etiolation in plants (Masuda et al., 2003). Studies have shown that POR proteins are required for catalyzing the light-dependent reduction of Pchlide to Chlide in plants (Reinbothe et al., 1999; Sakuraba et al., 2013). In the dark, POR and its substrate Pchlide are assembled into prolamellar bodies (PLBs) in etioplasts, and upon light exposure, photon is absorbed by the bound pigment and POR catalyzes the reduction of the C17-C18 double bond of the D-ring of Pchlide, leading to the production of Chlide (Heyes and Hunter, 2005; Blomqvist et al., 2008).

PORs are highly conserved proteins that are widely identified in angiosperms (Reinbothe et al., 2010). In *Arabidopsis*, there are three *POR* isoforms (*AtPORA*, *AtPORB*, and *AtPORC*) that display distinct developmental and light-regulated expression patterns (Armstrong et al., 1995; Masuda et al., 2003).*AtPORA* primarily presents in etiolated seedlings and its transcript level is rapidly decreased upon illumination (Armstrong et al., 1995). *AtPORB* is expressed in both etiolated seedlings and light-grown plants, while *AtPORC* is rarely detected in etiolated seedlings but is increased after several hours of light exposure (Masuda et al., 2003). Both *AtPORB* and *AtPORC* are required for bulk Chl synthesis throughout whole plant development in Arabidopsis (Paddock et al., 2010; Paddock et al., 2012). Under normal light conditions, neither *AtporB* nor *AtporC* single mutant exhibits obvious phenotypes, but *AtporB AtporC* double mutant displays seedling-lethal *xantha* phenotype at the cotyledon stage, suggesting that *AtPORB* and *AtPORC* function redundantly in the regulation of light-dependent Chl biosynthesis in Arabidopsis (Frick et al., 2003). In rice, there are only two *POR* isoforms, namely *OsPORA* and *OsPORB* (Kwon et al., 2017). The expression patterns of these two *OsPOR* genes are reminiscent of Arabidopsis *PORA* and *PORB* genes. *OsPORA* gene is mainly expressed in the dark during early leaf development, while *OsPORB* is expressed throughout the whole leaf growth stage independent of light signal. *faded green leaf* (*fgl*) mutant, which is disrupted in *OsproB* gene, displays faded green leaf, which is caused by the reduced Chl and carotenoid contents (Sakuraba et al., 2013). In addition, necrotic lesions occurring from the top area of older leaf blades are detected in the *fgl* mutant, which are largely caused by the increased accumulation of non-photoactive Pchlide and subsequently increased singlet oxygen upon illumination (Sakuraba et al., 2013). The association of Pchlide accumulation with singlet oxygen production has been well-elucidated in *flu* mutant in Arabidopsis. *flu* mutation leads to increased overaccumulation of Pchlide in darkness, which in turn results in a rapid elevation of singlet oxygen in chloroplasts upon illumination, and finally triggers cell death (Op Den Camp et al., 2003; Kim et al., 2012). In addition to Arabidopsis and rice, the function of *POR* genes has also been characterized in many other plant species, including barley (*Hordeum vulgare* L.) (Holtorf et al., 1995), wheat (*Triticum aestivum*) (Teakle and Griffiths, 1993), maize (*Zea mays*) (Selstam et al., 2002), tobacco (*Nicotiana tabacum* L.) (Selstam et al., 2002), and cabbage (*Brassica oleracea* cv. *Capitata*) (Nie et al., 2021).

In this study, we performed genetic screening for the mutants with reduced Chl content in quinoa, and a mutant with faded green leaf was identified. Based on bulk segregant analysis (BSA), we found that the faded green leaf phenotype was caused by a mutation in *CqPORB* gene. Further study revealed that disruption of *CqPORB* gene resulted in abnormal grana stacks and reduced conversion rate of photoactive Pchlide to Chlide upon light exposure. Expression patterns of *CqPOR* genes in response to light and in different developmental stages were also analyzed in this study.

## Materials and methods

### Plant materials and growth conditions

The quinoa variety NL-6 and Longli-4 were obtained from Prof. Heng Zhang and Prof. Pengshan Zhao, respectively. Quinoa seeds were directly sown in soil and kept at a growth chamber at 28°C under a light cycle of 12 h light/12 h dark. The phenotype of plants was observed and photographed after growth for indicated days. *Arabidopsis* Columbia (Col-0) ecotype was used for the transformation of *35S::CqPORB-GFP*. *Arabidopsis* seeds were sterilized and sown on half Murashige and Skoog (MS) solid medium and kept at 4°C for 3 days before being transferred to an incubator at 22°C with a long-day cycle (16 h light/8h dark).

### Ethyl methanesulfonate (EMS) mutagenesis in quinoa

For EMS treatment, a set of healthy seeds of quinoa NL-6 variety were presoaked in distilled water at 4°C for 8 h, and then the seeds were immersed in 1.0% EMS solution (v/v) (Sigma-Aldrich, M0880) and incubated at 4°C for 12 h. After treatment, a volume of 10% sodium thiosulphate (w/v) solution was added to the EMS solution, and the quinoa seeds were gently stirred and stand for 10 min before they were washed five times with distilled water to neutralize and remove EMS. The mutated seeds were sown in field for the collection of progeny seeds.

### Measurement of photosynthetic pigments

For Chl and carotenoid measurement, the leaves of four quinoa seedlings grown in the conditions of 28°C/26°C day/night temperatures and a 12 h/12 h light (216 µmol m^−2^·s^−1^)/dark cycle were sampled. Total pigments were extracted using 95% ethanol. The contents of Chl *a*, Chl *b* and carotenoid were measured using a NanoDrop™ 2000C UV-vis spectrophotometer (Thermo Scientific) as described previously (Lichtenthaler, 1987).

Total Pchlide and Chlide were calculated following the method described previously (Kwon et al., 2017). 20 seedlings were grown in darkness for 5 days or further exposed to white light for 10 s, 30 s, and 5 min. The seedlings were homogenized in 500 μL of ice-cold 80% acetone and incubated for 8 h in darkness (4°C). After spinning for 5 min, 150 μL of the supernatant was transferred to a 96-well plate (black, LABSELECT, 33113) that is made of polypropylene. Fluorescence was detected at an excitation wavelength of 440 nm, and emissions were collected from top between 600 nm and 720 nm at room temperature using a Thermo Scientific Varioskan Flash Plate Reader.

### Quinoa protoplast assay

The quinoa seeds were sown directly in soil and grown in a light incubator at 22°C (light)/20°C (dark) in long-day conditions (16 h light/8 h dark). The first or second pair of the true leaves of quinoa seedlings after growth for 15-20 days were collected using adhesive tape and dissociated in a dissociation solution (1.5% Cellulase R10, 0.4% Macerozyme R10, 0.5 M Mannitol, 20 mM KCl, 20 mM MES (pH 5.7), 10 mM CaCl_2_, and 0.1% BSA). The dissociation was carried out on a shaker with a speed of 50 rpm for 2.5 h under darkness at 22°C. The protoplasts were filtered through a Filter Mesh 100 and the filtered cells were centrifuged at 100 g for 3 min. The supernatant was removed and the cells were washed with 5-10 mL of W5 solution (154 mM NaCl, 125 mM CaCl_2_, 5 mM KCl, 2 mM MES (pH 5.7), and 0.1% (w/v) glucose) for three time with gentle shake. After washing, appropriate amount of MMg solution (0.4 M Mannitol, 15 mM MgCl_2_, and 4 mM MES (pH 5.7)) was added to adjust the cell concentration to about 2 × 10^5^ cells/mL. Subsequently, 100 μL protoplasts, 5 μL *CqPORB-GFP* plasmid (800-1000 ng/μL), and 105 μL 40% (w/v) PEG (0.2 M mannitol and 100 mM Ca(NO_3_)_2_) were slowly mixed, and the mixture was incubated at room temperature for 15 min. The transformation was terminated by adding 500 μL W5 solution. After centrifugation at 100 g for 3 min, the supernatant was discarded and protoplasts were washed twice with W5 solution. The protoplasts were transferred to cell culture plate and incubated at 22°C under low light conditions (5 µmol·m^−2^·s^−1^) for 36 h. Then protoplasts were collected and total Chl was extracted under light conditions, and Chl was measured according to the protocol described above.

### Transmission electron microscopy (TEM) analysis

The first true leaves of NL-6 and *nl6-35* harvested from 10-day-old seedlings grown in low-light conditions were used for the detection of granum in chloroplast. The samples were immersed in TEM fixative (G1102, Servicebio, China) containing 2.5% glutaraldehyde overnight at 4°C, and rinsed with 0.1 M sodium phosphate buffer (pH 7.4), and post-fixed with a 1% (w/v) aqueous solution of OsO_4_ at room temperature for 7 h. The materials were then dehydrated in a gradient ethanol series, and embedded in EMBed 812 resin (90529-77-4, SPI, USA). Tissues were processed into ultrathin sections, and stained with a 2% (w/v) uranium acetate and subsequently with 2.6% (w/v) lead citrate solution. The chloroplast was observed using a TEM device (HT7800, HITACHI, Japan).

### BSA-seq analysis of mutations in *nl6-35*

For BSA-seq analysis, *nl6-35* was backcrossed with its parent NL-6. In F_2_ population, 80 plants with faded green leaf were collected and pooled together for total DNA isolation using the DNeasy Plant Maxi kit (Qiagen). The library construction, sequencing, and bioinformatic analysis were all conducted by Personalbio (Shanghai, China). 150 bp pair-end sequencing libraries were generated and sequenced with an Illumina NovaSeq 6000 platform (Illumina lnc., San Diego, CA, USA). The clean reads that were filtered from raw reads were aligned to the reference genome of NL-6 using BWA (Li and Durbin, 2009). Picard 1.107 tools were used to convert alignment files to bam files and remove duplicated reads. GATK was applied to conduct local realignment around Indels and single-nucleotide polymorphism (SNP) calling (Zhu et al., 2014). Annovar program (Wang et al., 2010) was used to annotate all the SNPs based on the GFF3 files of the reference genome of NL-6. The SNP-index (Takagi et al., 2013) was calculated for all SNPs to identify candidate regions associated with the mutant trait, and the SNPs with a read depth ≥ 8 and SNP-index ≥ 0.3 were retained, and the average SNP-index across a 1-Mb genomic interval was measured individually using a 100-kb sliding-window approach and then plotted against all the chromosomes of quinoa genome. Finally, the SNPs with SNP-index = 1 were chosen for the manual analysis of candidate genes. To verify the SNP in *CqPORB* gene in *nl6-35* mutant, genomic sequence of *CqPORB* gene was amplified and sequenced using traditional Sanger sequencing. The gene-specific primers used for sequencing are listed in **Supplementary Table 1**.

### Molecular marker-associated analysis of *CqPORB* gene mutation in *nl6-35*

The identified SNP in *CqPORB* gene was used to design Kompetitive Allele Specific PCR (KASP) marker. The developed marker was used for the linkage analysis of the F_2_ population plants derived from a hybridization between *nl6-35* and its parent NL-6. Totally 93 plants with mutant phenotype and 95 plants with wild type phenotype were selected for KASP analysis. Besides, the mutant-type plants from the F_2_ progenies of Longli-4 × *nl6-35* crossing population were also selected for linkage analysis using the gene-specific KASP marker in *CqPORB* gene.

### Protein sequence alignment and phylogenetic analysis

Homologs of *CqPORB* were obtained by a Blastp search of GenomeNet (www.genome.jp), Plant GDB (www.plantgdb.org), and The Rice Annotation Project Database. Multiple protein sequences were aligned by ClustalW and conserved regions were visualized by CLC sequence viewer 8. The phylogenetic tree was constructed by iqtree with the best-fit model and 1,000 replicates of ultrafast bootstrap (Minh et al., 2020). The resulting tree files were submitted to iTOL (Letunic and Bork, 2021) for modification.

### Gene expression analysis

To analyze the expression pattern of *CqPORs* in response to light, surface sterilized seeds were sowed on ½ MS solid medium and incubated at 22°C for 3 days, and then etiolated seedlings were collected after exposure to white light for 0, 3, and 6 h. For the analysis of *CqPOR* gene expression in different developmental stages, the first true leaves of 10-day-old seedlings, leaves of early flowering stage (30 d), and late flowering stage (50 d) were sampled. Three independent replicates were performed. Total RNAs of all these samples were extracted by using EastepTM Super Total RNA Extraction Kit (Promega, United States). cDNAs were synthesized using HiScript III RT SuperMix for qPCR (+gDNA wiper) according to the manufacturer’s instructions (Vazyme, Nanjing, China). qRT-PCR was performed using ChamQ Universal SYBR qPCR Master Mix (Vazyme, Nanjing, China) and Bio-Rad CFX connect real time detection system (BIO-RAD, United States). Primers used for qRT-PCR are listed in **Supplementary Table 1**. The transcript levels of all three *CqPOR* genes in four different tissues were obtained from previous RNA-seq data (Jiang et al., 2022). Gene expression patterns of *CqPOR* genes in different tissues were visualized in heat map using TBtools software package (Minh et al., 2020).

### Plasmid construction and plant transformation

*CqPORB* was amplified using the cDNA template generated from the leaves of NL-6 using gene-specific primers (**Supplementary Table 1**). *CqPORB* was constructed into plant expression binary vector *pCAMBIA1305*, which carries a GFP reporter gene driven by the cauliflower mosaic virus (CaMV) 35S promoter. The recombinant plasmid *CqPORB-GFP* was introduced into the competent cells of *Agrobacterium tumefaciens* GV3101 strain, which was further used for transformation into *Nicotiana benthamiana* and *Arabidopsis thaliana* (Columbia). For transient expression in *N. benthamiana*, GV3101 strain harboring *CqPORB*-GFP was infiltrated into leaves according to the protocol described previously (Liu et al., 2010). Transformation of Arabidopsis plants was performed by using floral dip method (Clough and Bent, 1998). To detect the subcellular localization of *CqPORB*, GFP fluorescence was detected by Leica confocal laser scanning microscope SP8 with 488 nm excitation light and 500-550 nm emission light.

## Results

### *nl6-35* mutant exhibits reduced Chl

**Figure 1:**
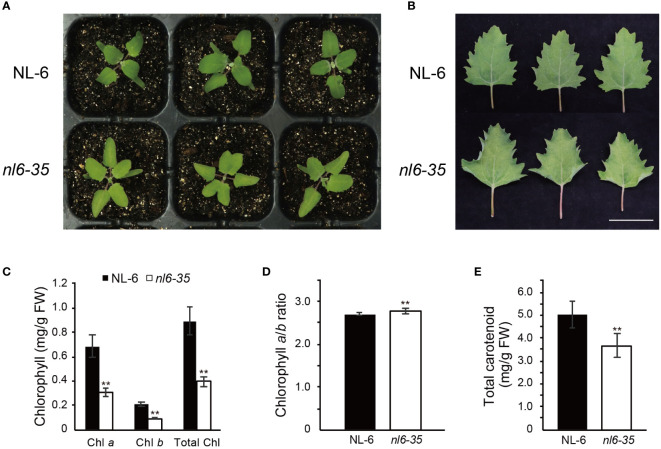
Identification of quinoa mutant *nl6-35* that exhibits faded green leaf and reduced Chl. **(A)** Phenotype of wild type (NL-6) and *nl6-35* plants grown on soils for 14 days under a light cycle of 12 h light/12 h dark. **(B)** Phenotype of the leaves of 30-day-old plants grown under a light cycle of 12 h light/12 h dark. Scale bar, 5 cm. **(C)** Measurement of Chl *a*, Chl *b*, and total Chl in wild type (NL-6) and *nl6-35* plants. Values are the means ± SD of six biological replicates. Asterisks indicate statistically significant differences (**p < 0.01, Student’s *t*-test). **(D)** The ratio of Chl *a*/Chl *b* in wild type (NL-6) and *nl6-35* plants. Values are the means ± SD of six biological replicates. Asterisks indicate statistically significant differences (**p < 0.01, Student’s *t*-test). **(E)** Quantification of total carotenoid content in each genotype. Values are the means ± SD of six biological replicates. Asterisks indicate statistically significant differences (**p < 0.01, Student’s *t*-test).

To investigate the regulatory mechanism of Chl biosynthesis in quinoa, we performed EMS-mediated mutagenesis for quinoa NL-6 variety and screened for mutants that displayed a reduced Chl content. NL-6 is a coastal germplasm that originates from lowland Chilean and exhibits relatively stable agronomic traits (Benlhabib et al., 2016). Here we report one of the mutants, designed *nl6-35*, that showed faded green leaf when grown in both greenhouse and field (**Figure 1A**, **Supplementary Figures 1A, B**). Specifically, we noted that at cotyledon stage, there was no significant difference between the wild type NL-6 and *nl6-35* (**Supplementary Figure 1C**), but the leaves of *nl6-35* became faded green after growth for approximately 14 days under white light (**Figure 1A**). Accompanying the faded green leaf, the phenotype of curled leaf was also observed in the *nl6-35* mutant, but not in the wild type NL-6 (**Figure 1B**). Genetic analysis indicated that the phenotypes of faded green leaf and curled leaf were tightly associated, suggesting that they are likely caused by a same mutation in the *nl6-35* mutant. Quantification of Chl content revealed that Chl *a* and Chl *b*, and total Chl contents were significantly reduced in the *nl6-35* mutant (**Figure 1C**), but the ratio of Chl *a*/Chl *b* was slightly increased in the *nl6-35* mutant (**Figure 1D**). In addition, the carotenoid content was also reduced by approximately 15.6% in the *nl6-35* (**Figure 1E**). These results suggested that the faded green leaf in *nl6-35* was likely caused by the reduced Chl and carotenoid contents.

### Identification of mutations in *nl6-35* by using BSA−seq

**Figure 2:**
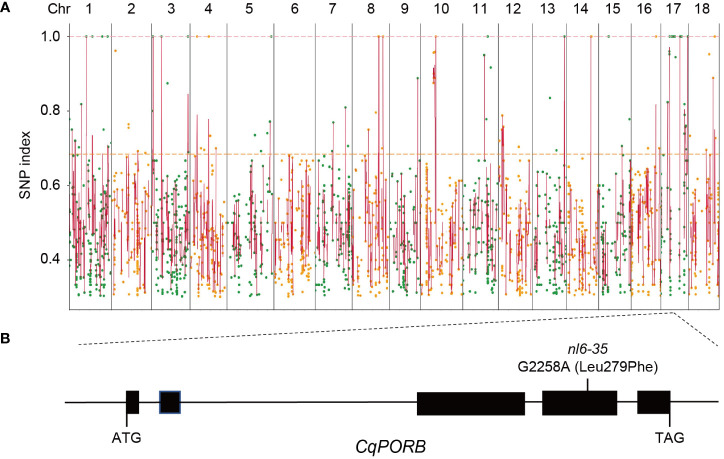
Identification of the mutated genes in *nl6-35* using BSA-seq analysis. **(A)** Identification of SNPs in *nl6-35* using the F_2_ hybridization population of the *nl6-35* mutant with its parent NL-6. Round dots indicate all the SNPs with a C to T or G to A change in the *nl6-35* mutant. X axis indicates the distribution of SNPs on all 18 chromosomes in quinoa. Y axis indicates the SNP index of each SNP. **(B)** Diagram illustrating the genomic structure of *CqPORB* gene. The G2258A mutation (Leu279phe) in *nl6-35* mutant is shown.

To identify the mutated gene in *nl6-35* that is responsible for the phenotype of faded green leaf, *nl6-35* was backcrossed with its parent NL-6. The F_1_ generation showed a wild type phenotype and F_2_ population exhibited a segregation of 3:1 (**Table 1**), indicating that *nl6-35* is a recessive mutation controlled by a single gene. To perform BSA-seq analysis, 80 plants of F_2_ population with faded green leaf were collected and mixed for whole genome sequencing. After filtering the raw reads, a total of 324,781,844 high quality reads were obtained, and 99.94% of the reads were mapped to NL-6 reference genome (1.39 GB), with an average of 34× reads coverage. Totally 11,960 SNPs were identified after removing the ones with low coverage (<8 reads). Because EMS mutagenesis most frequently causes C to T or G to A change in the genome, so we searched for these two types of nucleotide acid changes and finally obtained 3,013 SNPs with C to T or G to A change (**Figure 2A**, **Supplementary Figure 2**, **Supplementary Table 2**). Among these SNPs, 35 were identified with a mutation rate of 100%, and 15 of them were located on chromosome (chr)17 (**Supplementary Table 3**), suggesting that the objective gene is most likely located on chr17. Analysis of these 15 SNPs showed that 14 SNPs were located either in intronic or intergenic regions (**Supplementary Table 3**), while one SNP (G2258A) was identified in the fourth exon region of CqNL-6_047471, resulting in an amino acid change of Leu279 to Phe (**Figure 2B**). CqNL-6_047471 gene was annotated as a light-dependent protochlorophyllide reductase, which is homologous to *POR* genes reported in other plant species. It is worth noting that the faded green leaf of the *nl6-35* mutant is similar to the phenotype reported in *OsporB* mutant in rice (Kwon et al., 2017), suggesting that CqNL-6_047471 gene mutation is responsible for the phenotype of *nl6-35* mutant in quinoa. Because CqNL-6_047471 gene mutation resulted in a similar phenotype as the *OsporB* mutant in rice, we named CqNL-6_047471 as *CqPORB*.

**Table 1:** Phenotypic segregation in the F_2_ population of *nl6-35* × NL6 and Longli-4 × *nl6-35*.

F_2_ population	Wild type	Mutant type	χ2 for 3:1	*P* value
*nl6-35* × NL6	271	87	0.09	0.76
Longli-4 × *nl6-35*	298	83	2.10	0.14

### Validation of the association of *CqPORB* mutation with *nl6-35* mutant phenotype

Currently genetic transformation in quinoa is still technically limited, so we explored other strategies to verify the association of *CqPORB* gene mutation with the faded green leaf of *nl6-35*. In the F_2_ hybridization population of *nl6-35* with NL-6, we selected 93 plants that showed faded green leaf phenotype and 95 plants that showed wild type phenotype for the analysis of G2258A mutation in *CqPORB* gene. To perform genotyping in a high-throughput way, KASP (Kompetitive Allele Specific PCR) molecular marker that targeted this mutation site was designed. KASP analysis showed that in all 93 plants with mutant phenotype, the nucleotide acid G2258 was mutated to A, while in all 95 plants with wild type phenotype, G2258 site was either wild type or heterozygous, with a ratio of 27:68 (close to 1:2), verifying that *CqPORB* gene mutation is genetically associated with the mutant phenotype of *nl6-35* mutant.

In addition, we also crossed *nl6-35* mutant with another quinoa variety Longli-4 that originates from Bolivia. In F_2_ generation, the segregation of the faded green leaf phenotype is approximately 3:1 (**Table 1**), corroborating that the mutant phenotype in *nl6-35* was controlled by a recessive mutation. Similarly, 180 plants with faded green phenotype were selected for KASP analysis. The result showed that all these plants with mutant-type phenotype carried *CqPORB* allele that were derived from *nl6-35* but not from Longli-4 variety, which further supported that *CqPORB* gene mutation is associated with the faded green leaf of *nl6-35* mutant.

To further demonstrate that the reduced chlorophyll content in *nl6-35* mutant was caused by the *CqPORB* gene mutation, we generated protoplasts from both wild type NL-6 and *nl6-35* and transformed wild type *CqPORB* to the protoplasts of *nl6-35*. In protoplast, total Chl content in the *nl6-35* mutant was much lower than that of the NL-6. However, transformation of *CqPORB* gene, but not the empty vector, to the protoplasts of *nl6-35* significantly increased Chl content (**Supplementary Figure 3**), suggesting that *CqPORB* gene mutation is responsible for the reduced Chl content in the *nl6-35* mutant.

### *nl6-35* shows abnormal chloroplast development and reduced conversion of Pchlide to Chlide upon illumination

**Figure 3:**
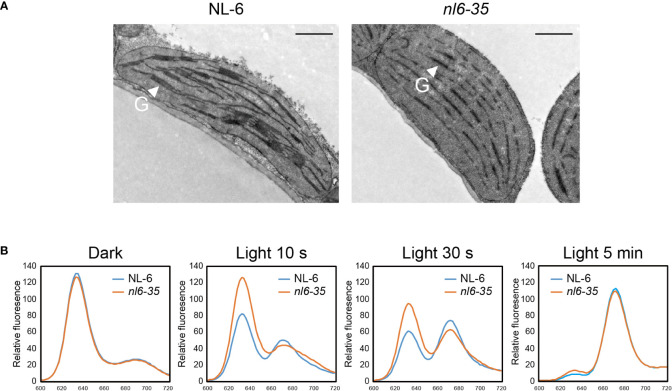
Transmission electron microscopy (TEM) analysis of plastids and measurement of Pchlide in *nl6-35* mutant. **(A)** 10-day-old wild type (NL-6) and *nl6-35* leaves grown under long-day conditions (16 h light/8 h dark) were collected for the analysis of grana in chloroplast. G, grana stacks. Scale bar, 1.0 μm. **(B)** Analysis of the conversion of Pchlide to Chlide upon illumination. Pigments were extracted from five-day-old etiolated seedlings in the dark or after being exposure to light for 10 s, 30 s, and 5 min. Total Pchlide was calculated from the fluorescence emission at 634 nm, while Chlide was calculated from the fluorescence emission at 672 nm.

Studies have shown that disruption of *POR* genes affects the development of thylakoids in both Arabidopsis and rice (Paddock et al., 2010; Paddock et al., 2012; Sakuraba et al., 2013). Here we took a close view of chloroplast in *nl6-35* by using transmission electron microscopy. In the wild type NL-6, numerous grana stacks were observed in the chloroplasts of light-grown plants. In the *nl6-35* mutant, however, only unstacked thylakoids were observed (**Figure 3A**), indicating that CqPORB is crucial for grana development in quinoa.

In Chl biosynthesis pathway, *POR* is an essential enzyme that catalyzes the reduction of Pchlide to Chlide in a light-dependent way (Tanaka and Tanaka, 2007). POR-bound Pchlide is termed ‘photoactive’ and be able to converted to Chlide instantaneously in the presence of light, whereas Pchlide not bound by POR is termed ‘non-photoactive’ and has been indicated as a factor causing photooxidative damage (Paddock et al., 2010). Here, we measured the content of total Pchlide and Chlide in NL-6 and *nl6-35* before and after light exposure for 10 s, 30s, and 5 min. Under dark conditions, the total Pchlide content was comparable between wild type NL-6 and *nl6-35*. However, after illumination, the photoactive Pchlide was reduced in a higher speed in NL-6 than in *nl6-35* mutant, while Chlide was increased in a higher rate in NL-6 than in *nl6-35* mutant, suggesting that *CqPORB* is important for the conversion of Pchlide to Chlide (**Figure 3B**).

### Characterization of *POR* genes in quinoa

The genomic length of *CqPORB* annotated in NL-6 genome was 2780 nucleotide acids, and this gene harbors five exons and encodes a protein of 395 amino acids. CqPORB shares high sequence similarities with its homologs in other plants species, including *Arabidopsis thaliana* At5g54190/PORA (77.5%), At4g27440/PORB (76.6%) and At1g03630/PORC (76.6%); *Oryzae sativa* Os04g0678700/PORA (70.3%) and Os10g0496900/PORB (72.8%); *Physcomitrella patens* Ppls146_112V6.1 (78.2%) and Ppls108_171V6.1 (68.2%); *Selaginella moellendorffii* SMpep_g142334 (71.0%); and *Chlamydomonas reinhardtii* Cre01.g015350.t1.1 (65.8%) (**Supplementary Figure 4**). This result suggested that POR proteins are conserved from algae to higher plants.

Alignment of CqPORB against the protein database of NL-6 genome revealed that there are two additional isoforms, CqNL-6_007045 and CqNL-6_022507, which are located on Chr4 and Chr12, respectively, based on the annotation of NL-6 genome. Quinoa is an allotetraploid plant that consists of A sub-genome and B sub-genome that originated from diploid *Chenopodium pallidicaule* and diploid *Chenopodium suecicum*, respectively (Jarvis et al., 2017). Based on synteny analysis of quinoa genome, it has been shown that Chr4 and Chr12 are the colinear chromosomes in quinoa that originated from the A and B sub-genomes of its progenitors (Jarvis et al., 2017). These clues suggest that CqNL-6_007045 and CqNL-6_022507 are evolutionarily the same isoform, and thereby they were designated as *CqPORA* (CqNL-6_007045) and *CqPORA-like* (CqNL-6_022507), respectively.

**Figure 4:**
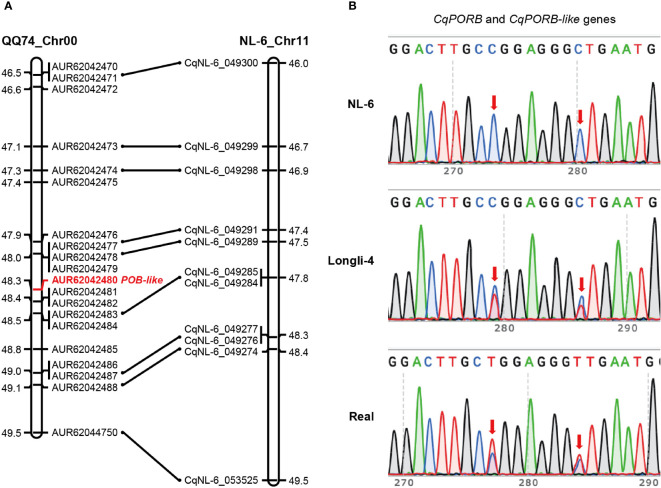
Collinearity of the *CqPORB-like* gene-located region between QQ74 and NL-6 varieties. **(A)** The *CqPORB-like* gene (AUR62042480) and its flanking genes in QQ74 variety were selected to align with the collinear chromosomal region in NL-6 variety. Note that *CqPORB-like* gene is lost in the NL-6 genome. **(B)** PCR amplification of *CqPORB* and *CqPORB-*like genes using a pair of primers matched to both genes in NL-6, Longli-4, and Real varieties. The PCR products were sequenced by sanger sequencing. Arrows indicate the variations between *CqPORB* and *CqPORB-like* genes.

Interestingly, we only identified one copy of *CqPORB* on Chr17 in NL-6 variety that originated from A sub-genome, indicating that the copy of *CqPORB* might be lost in the B sub-genome. To understand whether this is also the case in other quinoa varieties, we aligned CqPORB protein sequence with the protein database of the sequenced QQ74 variety (Jarvis et al., 2017). Unexpectedly, there are four *CqPOR* isoforms in QQ74 variety, and the two copies of *CqPORA* and *CqPORB* were identified in the A sub-genome and B sub-genome, respectively (**Supplementary Table 4**). These results indicated that the *CqPORB* copy was only lost in the NL-6, but not in QQ74. To understand whether *CqPORB-like* gene was truly lost in the genome of NL-6 or it was caused by the incomplete genome sequencing. The collinearity of the *CqPORB-like* gene and its flanking genes between QQ74 and NL-6 varieties was analyzed. Totally 20 genes, including *CqPORB-like* gene, were selected in the QQ74 genome, and among them 9 genes were also identified in the collinear region of NL-6 genome, but *CqPORB-like* gene and its neighboring genes was completely lost in the NL-6 genome (**Figure 4A**). To further exclude the possibility that the loss of *CqPORB-like* gene in NL-6 was caused by incomplete sequencing, primers that matched to both *CqPORB* and *CqPORB-like* genes were used for gene amplification using the genomic DNAs isolated from NL-6, Longli-4 and Real varieties, and PCR products were sequenced by sanger sequencing. The result showed that only one isoform was amplified in the NL-6 variety, but two isoforms were amplified in both Longli-4 and Real varieties (**Figure 4B**), verifying that *CqPORB-like* gene was lost in the NL-6 genome.

### Gene expression analysis of *CqPORs* in quinoa

**Figure 5:**
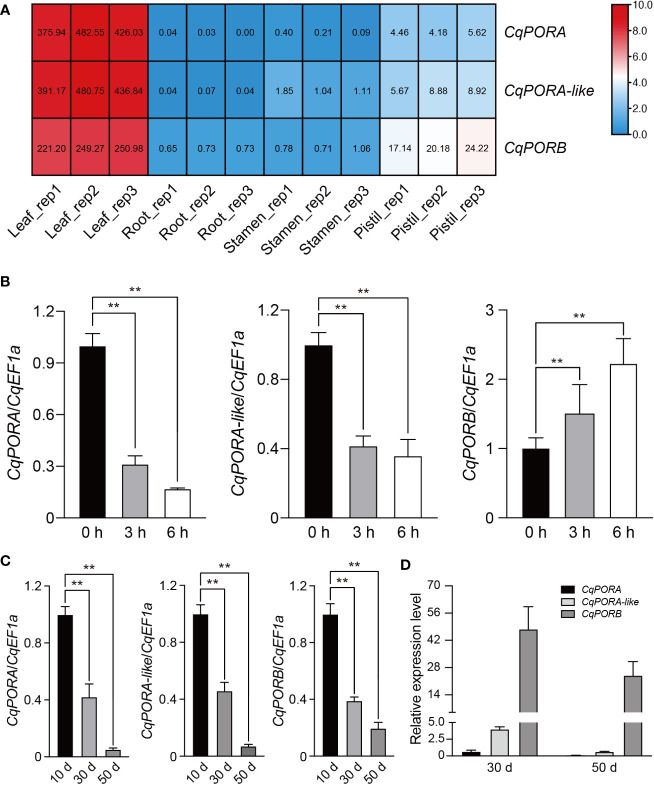
Expression patterns of *POR* genes in quinoa. **(A)** RNA-seq assay was performed for the leaf, root, stamen, and pistil tissues of NL-6 variety, and three independent replicates were conducted for each tissue. Different colors in the heatmap indicate the relative expression level of each *CqPOR* gene in different tissues based on log_2_-transformed FPKM. The FPKM values of *CqPOR* genes in four tissues are shown. **(B)** qRT-PCR analysis of the expression level of each *CqPOR* gene in etiolated seedlings after being exposed to white light for 0, 3, and 6 h. *CqEF1a* was used as an internal control. The transcript level of each *CqPOR* gene at 0 h was set as 1. Values are the means ± SD of three biological replicates. Asterisks indicate statistically significant differences (**p < 0.01, Student’s *t*-test). **(C)** qRT-PCR-analysis of the expression level of each *CqPOR* gene in NL-6 after growth on soils for 10, 30, and 50 days. *CqEF1a* was used as an internal control. The transcript level of each *CqPOR* gene at 10 d was set as 1. Values are the means ± SD of three biological replicates. Asterisks indicate statistically significant differences (**p < 0.01, Student’s *t*-test). **(D)** Comparison of the relative expression levels of the three *CqPOR* genes in NL-6 after growth on soils for 30 and 50 days. Values are the means ± SD of three biological replicates.

In a previous study, we have performed RNA-seq analysis for four different tissues, including leaf, root, stamen, and pistil in NL-6 variety (Jiang et al., 2022). Using these transcriptomic data, we found that all the three *CqPOR* genes were highly expressed in the leaves, but with a much less level in pistil, and they were almost not detected in root and stamen (**Figure 5A**). This gene expression pattern is consistent with the role of *POR* genes in the regulation of Chl biosynthesis in photosynthetic tissues. Moreover, the transcriptomic data showed that *CqPORA* and *CqPORA-like* genes rather than *CqPORB* gene exhibited a more similar gene expression pattern (**Figure 5A**).

It has been known that the expression of *POR* genes is altered upon illumination (Armstrong et al., 1995). Here, we tested the expression of these three *CqPOR* genes in NL-6 etiolated seedlings during the transit from dark to white light. qRT-PCR analysis showed that both *CqPORA* and *CqPORA-like* genes were down-regulated, while *CqPORB* gene was up-regulated after exposure to light (**Figure 5B**). The similar expression pattern of *CqPORA* and *CqPORA-like* genes in response to light further supported that they are evolutionarily the same isoform. The different expression pattern between *CqPORA* and *CqPORB* genes indicated that these two genes may exhibit a distinct function in the regulation of Chl synthesis in response to ambient light signal. We also analyzed the expression of these three *POR* genes in different developmental stages in quinoa, and found that after growth on soils for 10, 30, and 50 d, the expression levels of all these three *POR* genes were gradually decreased, but the *CqPORB* gene was reduced in a slower rate than the *CqPORA* genes (**Figure 5C**). At later developmental stages, we noted that the transcript level of *CqPORB* was much higher than the *CqPORA* and *CqPORA-like* genes (**Figure 5D**), indicating that *CqPORB* plays a predominant role in Chl biosynthesis and chloroplast development in mature plants.

### *CqPORB* is localized in chloroplast

**Figure 6:**
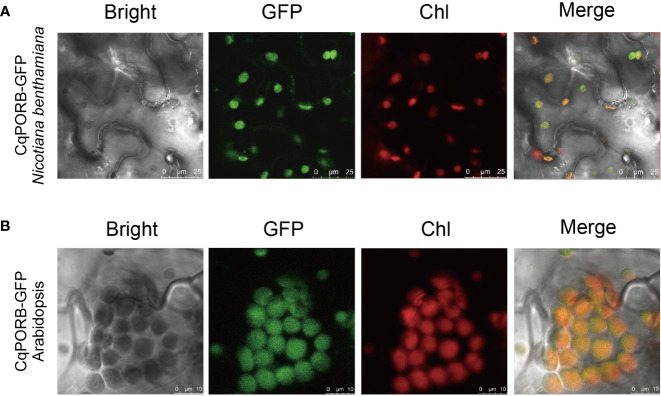
Analysis of the subcellular localization of CqPORB. **(A)** *CqPORB-GFP* was transiently expressed in *Nicotiana benthamiana*, and fluorescence was detected using confocal laser scanning microscopy. Scale bar, 25 µm. **(B)** The subcellular localization of CqPORB was analyzed in Arabidopsis transgenic plants expressing CqPORB-GFP. Fluorescence was detected using confocal laser scanning microscopy. Scale bar, 10 µm.

To study the subcellular localization of CqPORB in quinoa, we infiltrated *Agrobacterium* strain harboring *35S::CqPORB-GFP* plasmid into quinoa leaves according to the protocol described previously (Xiao et al., 2022). However, after transformation for 48 h, no GFP fluorescence signal was detected in quinoa leaves, suggesting that the efficiency of transient gene expression in quinoa leaves is still very low. We then transformed *35S::CqPORB-GFP* plasmid to tobacco leaves, and found that CqPORB was exclusively localized in chloroplast (**Figure 6A**). Besides, *35S::CqPORB-GFP* was transformed into Arabidopsis to obtain stable transgenic plants, and in these transgenic plants, GFP fluorescence was also clearly observed in chloroplast (**Figure 6B**).

## Discussion

Quinoa is an allotetraploid plant that harbors two similar genomes (Jarvis et al., 2017), so genetic study of a specific gene function in quinoa is a challenging work. However, using EMS-mediated mutagenesis of NL-6 variety, we identified some mutants with altered phenotypes, including early flowering, dwarfism, yellow leaves, and branched plants, suggesting that some phenotypes of quinoa are control by a single gene derived from A sub-genome or B sub-genome of quinoa or the biological functions of two gene copies originated from progenitors diverged during evolution. Based on BSA-seq analysis, 3,013 SNPs that were derived from C to T or G to A change were identified in *nl6-35* mutant, and a region containing candidate mutated genes was clearly detected, indicating that EMS-mediated mutagenesis is effective in quinoa, and whole genome sequencing-based mapping can be efficiently used to identify mutated genes in quinoa.

In this study, we report that mutation of *CqporB* gene alone leads to faded green leaf phenotype, which is mainly caused by the reduced Chl and carotenoid contents. Homolog analysis showed that only one copy of *CqPORB* that locates in A sub-genome was identified in NL-6, implying that *CqPORB* copy in B sub-genome was lost during evolution, and this could be one of the reasons that we could identify *CqporB* mutant based on EMS mutagenesis-mediated approach. It has been reported that, compared to other quinoa varieties, NL-6 variety exhibits yellow panicles and relatively reduced biomass (Benlhabib et al., 2016), but the molecular mechanisms underlying these phenotypes are still unknown. Based on our data, we can speculate that these phenotypes may be caused by the existence of only one copy of *CqPORB* in the genome of NL-6 variety. In future, the reasons underlying the loss of *CqPORB-like* gene in NL-6 but not in other quinoa varieties worth further investigation.

The function of PORs has been studied in many different plant species, including Arabidopsis, rice, wheat, and barley (Teakle and Griffiths, 1993; Armstrong et al., 1995; Holtorf et al., 1995; Sakuraba et al., 2013). The number of *POR* genes vary in different plant species. In Arabidopsis, there are three *POR* genes, *AtPORA*, *AtPORB*, and *AtPORC*, and these three genes exhibit distinct expression patterns in response to light signal (Armstrong et al., 1995). In rice, only two *POR* genes, *OsPORA* and *OsPORB*, were identified, and their expression levels were both altered after illumination, despite in an opposite way (Sakuraba et al., 2013). Similar to rice, there are two *POR* isoforms in quinoa, and these two isoforms also exhibited an opposite response to light, with *CqPORA* down-regulated and *CqPORB* up-regulated after light exposure, suggesting that these two *POR* genes are conserved in terms of gene expression pattern in different plant species. Our result showed that *nl6-35* mutant, in which *CqPORB* gene is mutated, did not exhibit obvious phenotypes in young seedling stage compared with the wild type, but all mutant plants display faded green leaf after growth for approximately 14 days. This phenomenon can be explained by the fact that both *CqPORA* and *CqPORB* genes are expressed in young seedings, but in mature plants, only *CqPORB* gene is predominately expressed. In line with the role of POR proteins in the biosynthesis of Chl, CqPORB is exclusively localized in the chloroplast, and RNA-seq data showed that all three *CqPOR* genes in NL-6 variety were highly expressed in leaves, but much less or not expressed in root, stamen, and pistil.

*CqPORB* gene encodes a NADPH:protochlorophyllide oxidoreductase that is required for the photo-reduction of Pchlide to Chlide (Heyes and Hunter, 2005). Recently, the structure-based mechanism of the reduction of Pchlide to Chlide upon illumination has been addressed. Plant POR is able to bind to Pchlide, NADPH, and lipids to forms helical filaments around lipid bilayer tubes, which facilitates the target of its product, Chlide, to the membrane for chlorophyll biosynthesis (Nguyen et al., 2021). Our result showed that the conversion of photoactive Pchlide to Chlide was disrupted in *CqporB* mutant in quinoa, which is similar to that observed in *OsporB* mutant in rice (Sakuraba et al., 2013), suggesting that PORB proteins in different plant species exhibit a similar function. Our data also showed that *CqporB* gene mutation resulted in abnormal grana stacks, which is similar to that of *AtporB AtporC* double mutant in Arabidopsis and that of *OsporB* mutant in rice (Paddock et al., 2010; Sakuraba et al., 2013), demonstrating that POR proteins play a structural role in the formation of grana stacks in chloroplast. In rice, it has been shown that disruption of *CqporB* gene results in necrotic lesions in leaves, which is largely caused by the increased accumulation of non-photoactive Pchlide and increased production of singlet oxygen (Sakuraba et al., 2013). In future, the production of singlet oxygen in *CqporB* mutant upon illumination is worth investigating. In addition, the role of FLU protein in the regulation of Pchlide accumulation and singlet oxygen production in quinoa needs to be further explored.

Quinoa has been regarded as a nutritional crop to contribute to global food security, and the demand for quinoa is expected to be rapidly increased in near future (Bazile et al., 2016). Because of the importance of quinoa as nutritional food, governments and companies in many countries have put increasing efforts to expand quinoa cultivation in non-native areas and improve the yield of quinoa. Due to some limited agronomic traits, large-scale cultivation of quinoa in non-native areas is still limited (Mizuno et al., 2020), so breeding of quinoa varieties with improved agronomic traits that adapt to diverse climate and soil conditions becomes increasingly urgent. Taking advantage of the high-quality quinoa reference genome and diverse quinoa varieties, molecular marker-assisted breeding will be an effective way to cultivate quinoa with improved agronomic traits. Photosynthesis is often explored as a target for the crop yield improvement (Long et al., 2015; Walter and Kromdijk, 2022), so identification of genes involved in photosynthesis in quinoa will provide valuable genetic loci for yield improvement of this pseudocereal crop.

## Data availability statement

Publicly available datasets were analyzed in this study. This data can be found here: NCBI, GSE198572.

## Author contributions

CL, MR, and CZ contributed to conception and design of the study. CL, MR, JL, and XW performed the most of experiments. QW and HZ conducted protein subcellular localization analysis. QZ, JY, and FY provided quinoa materials and assisted the growth of quinoa. HZ and J-KZ participated in scientific discussions. CZ wrote the manuscript with contributions from CL and MR. All authors contributed to the article and approved the submitted version.

## Supplementary material

Supplementary Figure 1 The growth phenotype of *nl6-35* mutant. **(A)** Phenotype of wild type (NL-6) and *nl6-35* plants grown in a growth chamber for 40 days. Scale bar, 10 cm. **(B)** Phenotype of wild type (NL-6) and *nl6-35* plants grown in field for 80 days.**(C)** Cotyledons of wild type (NL-6) and *nl6-35* seedlings grown on soils for 7 days. Scale bar, 1 cm.

Supplementary Figure 2 Identification of SNPs in *nl6-35* mutant using BSA-seq. The F_2_ plants of the crossing population of *nl6-35* and NL-6 were collected for whole-genome sequencing. All SNPs with C to T or G to A change were selected and distributed on the chromosomes of quinoa.

Supplementary Figure 3 Transformation of *CqPORB* gene rescues the reduced Chl content in *nl6-35* mutant using protoplast system. Protoplasts from wild type NL-6 and *nl6-35* were generated, and *CqPORB* gene was transformed into the protoplasts of *nl6-35* before total Chl was measured. The protoplasts transformed with empty vector were used as control. Values are the means ± SD of three biological replicates. Asterisks indicate statistically significant differences (**p < 0.01, Student’s t-test).

Supplementary Figure 4 Alignment of POR homologs in different plant species. **(A)** Alignment of POR proteins in *Chenopodium quinoa*, *Arabidopsis thaliana*, *Oryzae sativa*, *Physcomitrella patens*, *Selaginella moellendorffii*, and *Chlamydomonas reinhardtii*. **(B)** Phylogenetic analysis of PORs in different plant species. Phylogenetic tree was generated by using IQ-TREE2 program.
