# Supplementary figures and images for: Functional analysis of CqPORB in the regulation of chlorophyll biosynthesis in Chenopodium quinoa
Source: Front Plant Sci. 2022 Dec 12;13:1083438. doi: 10.3389/fpls.2022.1083438 (PMC9791128; doi:10.3389/fpls.2022.1083438)

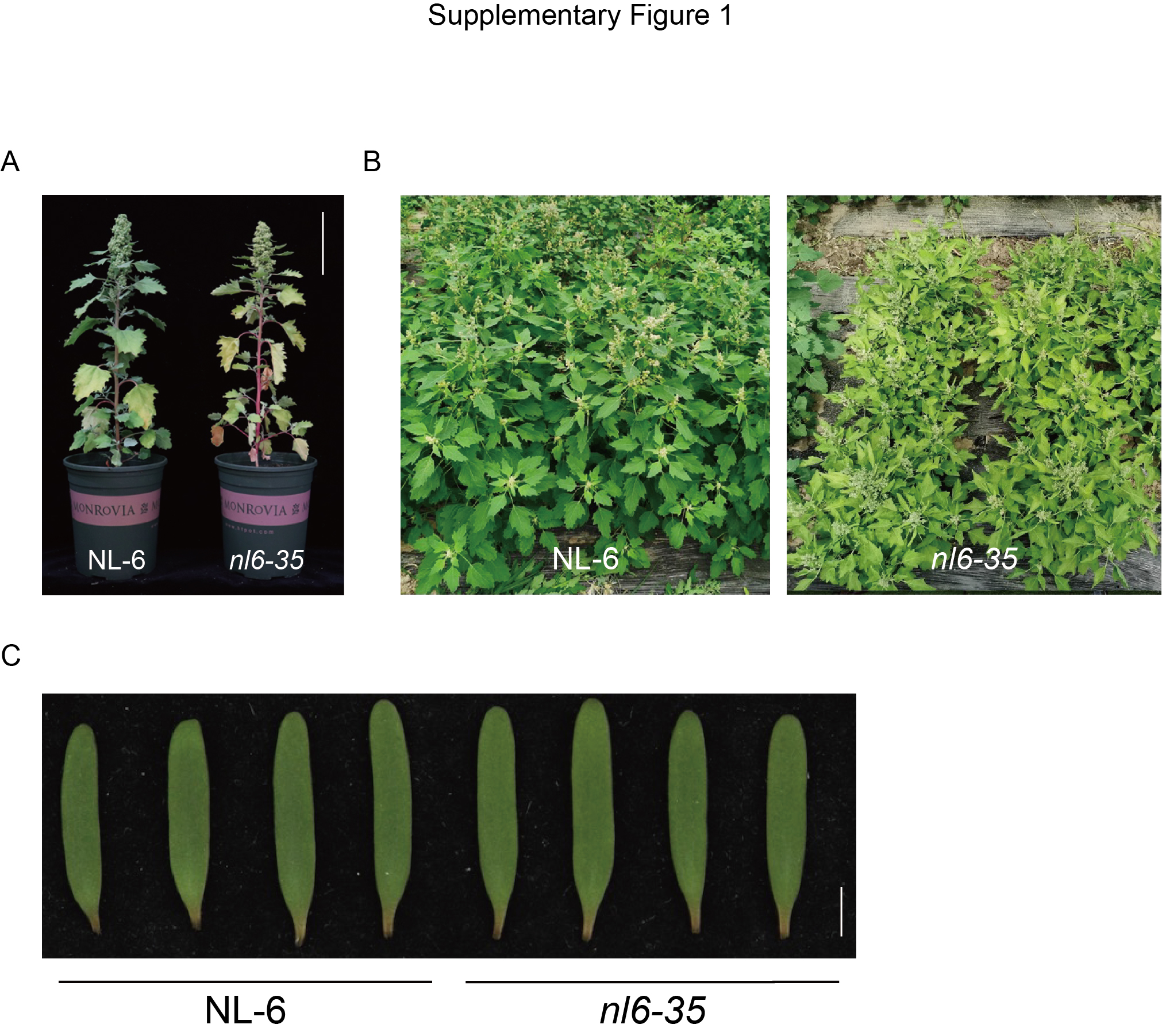

Supplement: Supplementary Figure 1 — The growth phenotype of nl6-35 mutant. (A) Phenotype of wild type (NL-6) and nl6-35 plants grown in a growth chamber for 40 days. Scale bar, 10 cm. (B) Phenotype of wild type (NL-6) and nl6-35 plants grown in field for 80 days.(C) Cotyledons of wild type (NL-6) and nl6-35 seedlings grown on soils for 7 days. Scale bar, 1 cm. [file Image_1.jpeg]

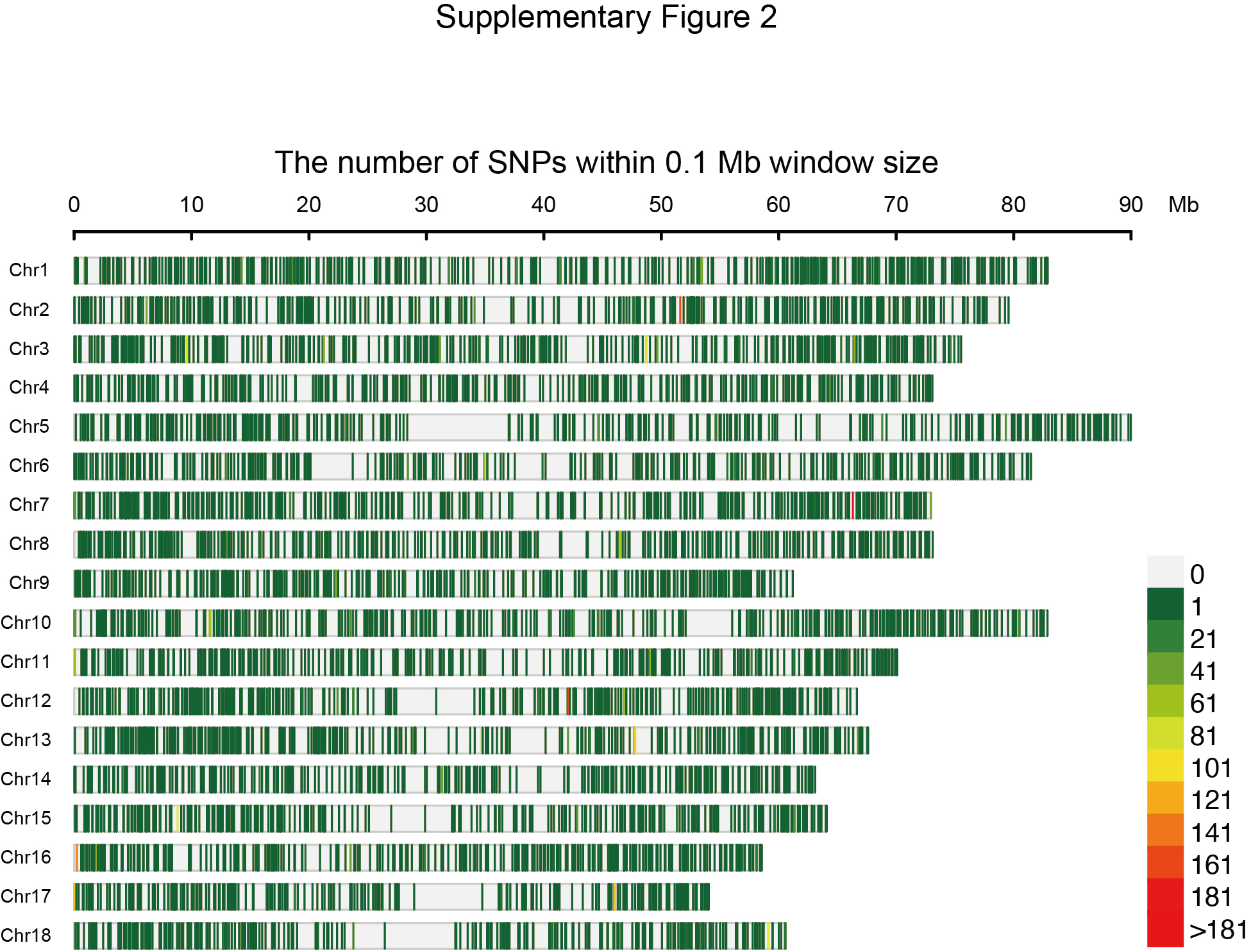

Supplement: Supplementary Figure 2 — Identification of SNPs in nl6-35 mutant using BSA-seq. The F2 plants of the crossing population of nl6-35 and NL-6 were collected for whole-genome sequencing. All SNPs with C to T or G to A change were selected and distributed on the chromosomes of quinoa. [file Image_2.jpeg]

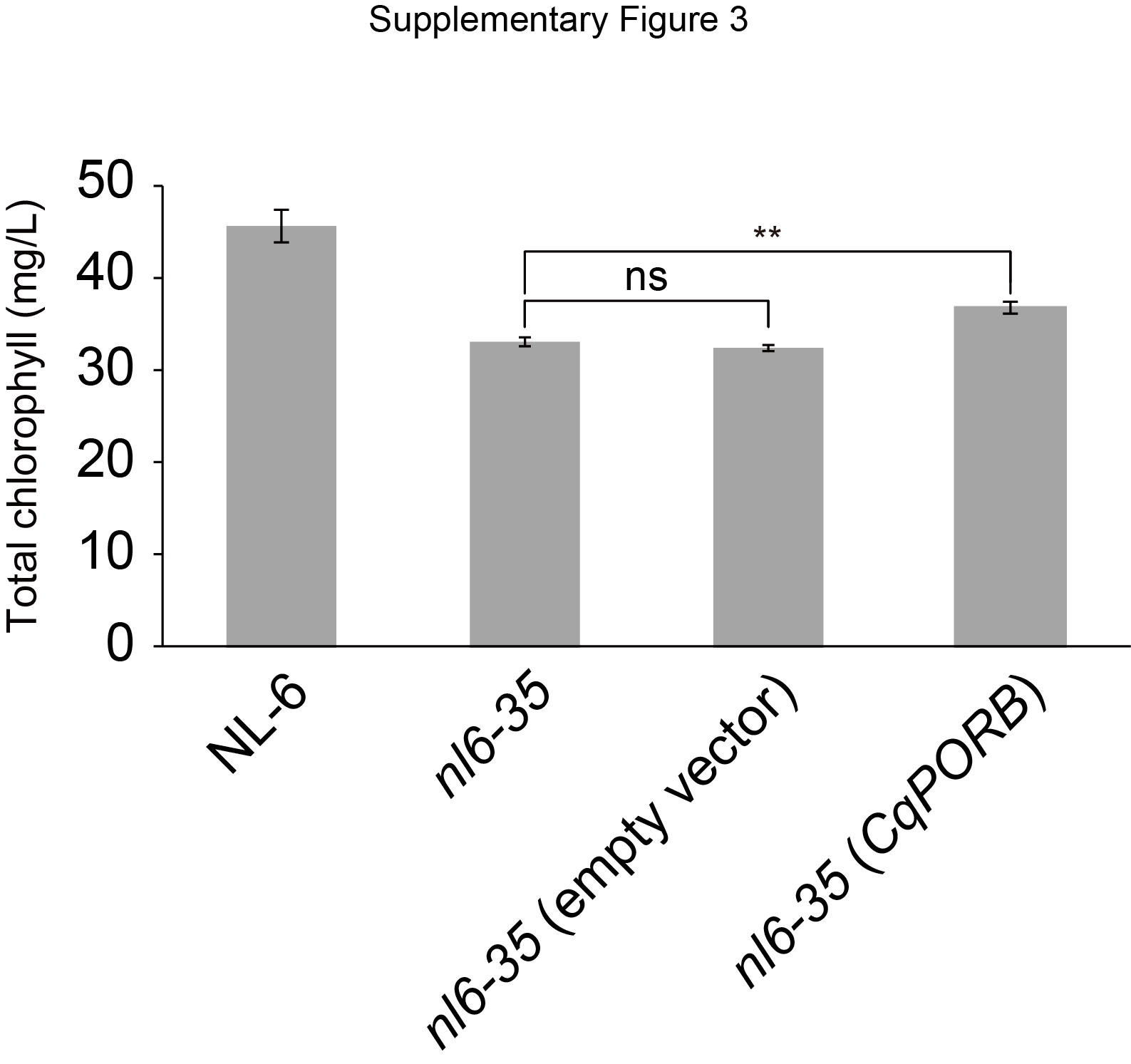

Supplement: Supplementary Figure 3 — Transformation of CqPORB gene rescues the reduced Chl content in nl6-35 mutant using protoplast system. Protoplasts from wild type NL-6 and nl6-35 were generated, and CqPORB gene was transformed into the protoplasts of nl6-35 before total Chl was measured. The protoplasts transformed with empty vector were used as control. Values are the means ± SD of three biological replicates. Asterisks indicate statistically significant differences (**p < 0.01, Student’s t-test). [file Image_3.jpeg]

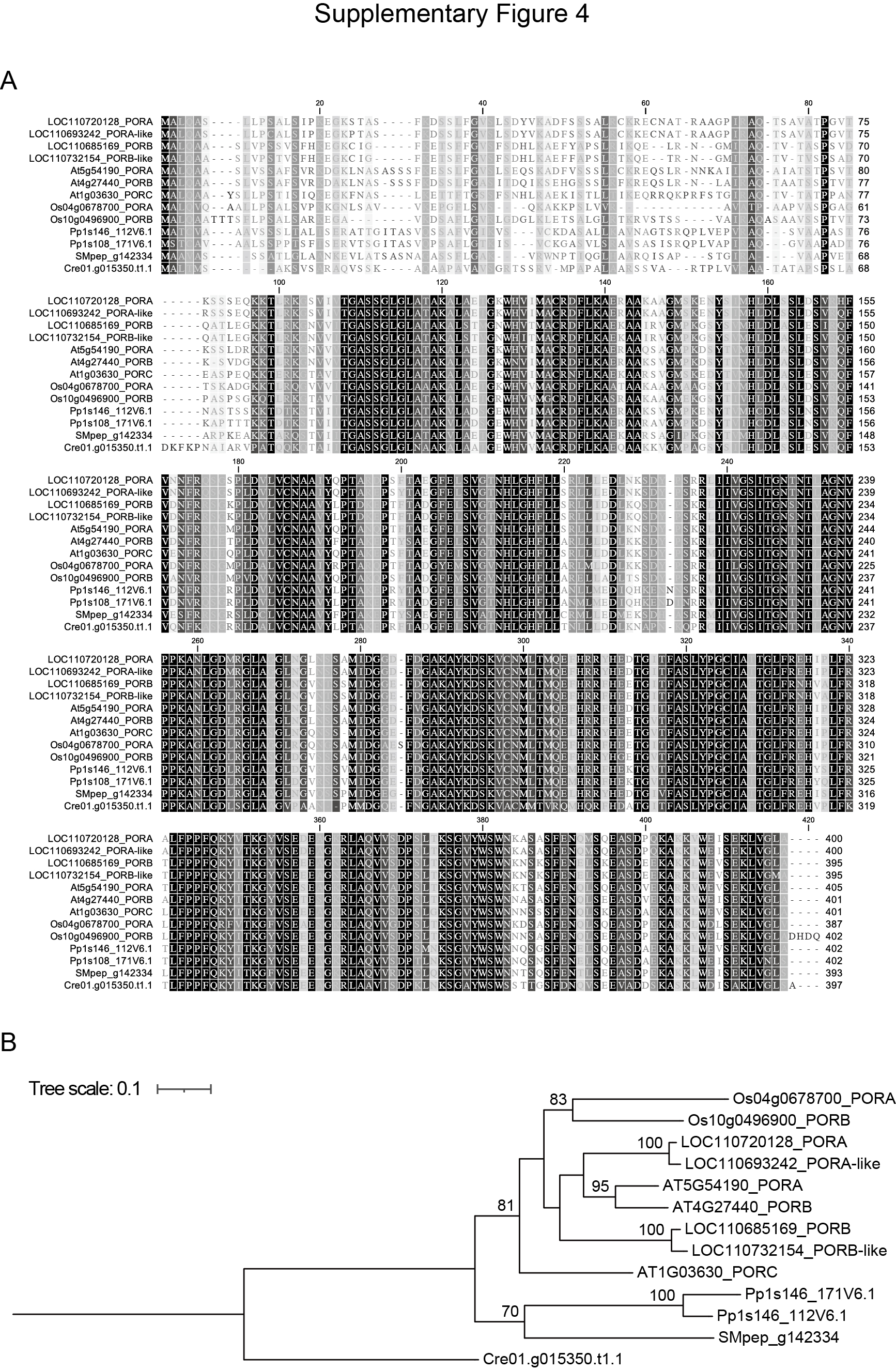

Supplement: Supplementary Figure 4 — Alignment of POR homologs in different plant species. (A) Alignment of POR proteins in Chenopodium quinoa, Arabidopsis thaliana, Oryzae sativa, Physcomitrella patens, Selaginella moellendorffii, and Chlamydomonas reinhardtii. (B) Phylogenetic analysis of PORs in different plant species. Phylogenetic tree was generated by using IQ-TREE2 program. [file Image_4.jpeg]
