# Supplementary material for: Functional analysis of CqPORB in the regulation of chlorophyll biosynthesis in Chenopodium quinoa
Source: Front Plant Sci. 2022 Dec 12;13:1083438. doi: 10.3389/fpls.2022.1083438 (PMC9791128; doi:10.3389/fpls.2022.1083438)
Supplement: Supplementary file 5 [file Table_1.docx]

Supplementary Table 1. Primers used for qPCR analysis, sequencing and plasmid construction

| Primer Name | Sequnces (5’-3’) | |
| --- | --- | --- |
| For qRT-PCR analysis of the expression of *CqPOR* genes | | |
| CqPORA_F | CACTTTAACATTACATTTCACTCAAGAC | |
| CqPORA_R | TAGGGATGGACAAGGCAG | |
| CqPORA-like_F | AAACAGCAAGTTCTTAATTTGG | |
| CqPORA-like_R | ATGCAGTAGATTTTCCCTCC | |
| CqPORB_F | CAGCAGCCAAGTATCCAAC | |
| CqPORB_R | TATACACTGCACTGTTTCCTTC | |
| For sequencing of the mutation site of the *CqPORB* gene | | |
| *nl6-35*_snp_F | GATAACTTTCGCCGTTCTGG | |
| *nl6-35*_snp_R | AGCCTATTTCCAGCCTCCTC | |
| For amplification of the genomic region of *CqPORB* and *CqPORB-like* genes | | |
| CqPORBg_F | CCTACCTTTACTGCTGATGG | |
| CqPORBg_R | CAGCTACTGGGGTACAACTC | |
| For construction of plasmid overexpressing *CqPORB* | | |
| CqPORBox_F | AGAACACGGGGGACGAGCTCATGGCGCTACAGGCTGCATC |  |
| CqPORBox_R | AGGTCGACTCTAGAGGATCCAGCCAATCCAACAAGTTTTTCG |  |
