# Supplementary material for: Functional analysis of CqPORB in the regulation of chlorophyll biosynthesis in Chenopodium quinoa
Source: Front Plant Sci. 2022 Dec 12;13:1083438. doi: 10.3389/fpls.2022.1083438 (PMC9791128; doi:10.3389/fpls.2022.1083438)
Supplement: Supplementary file 7 [file Table_3.docx]

| **Name** | **GeneID** | **Chrom** | **Position** | **Ref.** | **Alt.** | **Region** |  |
| --- | --- | --- | --- | --- | --- | --- | --- |
| **SNPs on Chr 17** | |  |  |  |  |  |  |
| SNP1 | CqNL-6_039906-CqNL-6_039905 | Chr17 | 17072840 | C | T | intergenic |  |
| SNP2 | CqNL-6_041906 | Chr17 | 21318945 | C | T | intronic |  |
| SNP3 | CqNL-6_041893-CqNL-6_041892 | Chr17 | 21694298 | C | T | intergenic |  |
| SNP4 | CqNL-6_041844-CqNL-6_041843 | Chr17 | 23600440 | C | T | intergenic |  |
| SNP5 | CqNL-6_041823-CqNL-6_041822 | Chr17 | 24556964 | C | T | intergenic |  |
| SNP6 | CqNL-6_041820-CqNL-6_041819 | Chr17 | 24773508 | C | T | intergenic |  |
| SNP7 | CqNL-6_041798-CqNL-6_041797 | Chr17 | 25633170 | C | T | intergenic |  |
| SNP8 | CqNL-6_041776-CqNL-6_041775 | Chr17 | 27338371 | C | T | intergenic |  |
| SNP9 | CqNL-6_041770-CqNL-6_041769 | Chr17 | 27571924 | C | T | intergenic |  |
| SNP10 | CqNL-6_047471 | Chr17 | 37710709 | G | A | exonic |  |
| SNP11 | CqNL-6_047477-CqNL-6_047478 | Chr17 | 38156169 | G | A | intergenic |  |
| SNP12 | CqNL-6_047507 | Chr17 | 39387211 | G | A | intronic |  |
| SNP13 | CqNL-6_047512-CqNL-6_047513 | Chr17 | 39758868 | G | A | intergenic |  |
| SNP14 | CqNL-6_047527-CqNL-6_047528 | Chr17 | 40988983 | G | A | intergenic |  |
| SNP15 | CqNL-6_031579-CqNL-6_031578 | Chr17 | 53022999 | G | A | intergenic |  |
| **SNPs on other Chrs** | |  |  |  |  |  |  |
| _chr1_SNP1 | CqNL-6_048039 | Chr1 | 33566184 | G | A | intronic |  |
| _chr1_SNP2 | CqNL-6_051625-CqNL-6_051626 | Chr1 | 45414165 | G | A | intergenic |  |
| _chr1_SNP3 | CqNL-6_036310-CqNL-6_036309 | Chr1 | 65075184 | C | T | intergenic |  |
| _chr1_SNP4 | CqNL-6_016397 | Chr1 | 75573297 | G | A | intergenic |  |
| _chr3_SNP1 | CqNL-6_051056 | Chr3 | 2360439 | C | T | intergenic |  |
| _chr3_SNP2 | CqNL-6_008354 | Chr3 | 19234919 | C | T | intronic |  |
| _chr3_SNP3 | CqNL-6_046181 | Chr3 | 71520183 | C | T | intergenic |  |
| _chr4_SNP1 | CqNL-6_007328-CqNL-6_007329 | Chr4 | 13831213 | G | A | intergenic |  |
| _chr4_SNP2 | CqNL-6_054258-CqNL-6_058417 | Chr4 | 36980863 | G | A | intergenic |  |
| _chr5_SNP1 | CqNL-6_026822-CqNL-6_026823 | Chr5 | 87204323 | G | A | intergenic |  |
| _chr8_SNP1 | CqNL-6_052925-CqNL-6_052924 | Chr8 | 51987664 | G | A | intergenic |  |
| _chr8_SNP2 | CqNL-6_035562-CqNL-6_035561 | Chr8 | 60694010 | G | A | intergenic |  |
| _chr10_SNP1 | CqNL-6_030247-CqNL-6_030248 | Chr10 | 29622721 | G | A | intergenic |  |
| _chr11_SNP1 | CqNL-6_049274-CqNL-6_049273 | Chr11 | 48829259 | C | T | intergenic |  |
| _chr13_SNP1 | CqNL-6_020813-CqNL-6_020814 | Chr13 | 63348717 | G | A | intergenic |  |
| _chr14_SNP1 | CqNL-6_029640 | Chr14 | 48061886 | C | T | intronic |  |
| _chr14_SNP2 | CqNL-6_029583-CqNL-6_029582 | Chr14 | 49097597 | G | A | intergenic |  |
| _chr15_SNP1 | CqNL-6_054535 | Chr15 | 19782992 | C | T | exonic |  |
| _chr16_SNP1 | CqNL-6_002059 | Chr16 | 49183250 | G | A | intronic |  |
| _chr18_SNP1 | CqNL-6_031651 | Chr18 | 51832881 | G | A | intronic |  |

Supplementary Table 3. List of SNPs on Chr17.
