# Supplementary material for: Functional analysis of CqPORB in the regulation of chlorophyll biosynthesis in Chenopodium quinoa
Source: Front Plant Sci. 2022 Dec 12;13:1083438. doi: 10.3389/fpls.2022.1083438 (PMC9791128; doi:10.3389/fpls.2022.1083438)
Supplement: Supplementary file 8 [file Table_4.docx]

| Gene Name | Gene ID in QQ74 | mRNA | Predicted protein (aa) | Chr | Gene ID in NL-6 | mRNA | Predicted protein (aa) | Chr |  |
| --- | --- | --- | --- | --- | --- | --- | --- | --- | --- |
|  |  |  |  |  |  |  |  |  |  |
| *PORA* | LOC110720128 | XM_021899131.1 | 400 | Chr05 | CqNL-6_007045 | CqNL-6_007045.1 | 400 | Chr04 |  |
| *PORA-like* | LOC110693242 | XM_021870397.1 | 400 | Chr12 | CqNL-6_022507 | CqNL-6_022507.1 | 400 | Chr12 |  |
| *PORB* | LOC110685169/ AUR62037335 | XM_021861627.1/ AUR62037335 | 395 | Chr15 | CqNL-6_047471 | CqNL-6_047471.1 | 395 | Chr17 |  |
| *PORB-like* | LOC110732154/ AUR62042480 | XM_021912068.1 | 395 | Chr00 | — | — | — | — |  |

Supplementary Table 4. Physical positions of *CqPORs* in the reference genome of QQ74 and NL-6.
